# Outpatient utilization trend of bronchodilator and anti-inflammatory agents in the pandemic and beyond

**DOI:** 10.1007/s00210-025-04099-7

**Published:** 2025-04-08

**Authors:** N. Ipek Kirmizi Sonmez, Onur Gultekin, Ahmet Akici, Yelda Basbug, Volkan Aydin

**Affiliations:** 1 Department of Pharmacology, School of Pharmacy, Bahcesehir University, Istanbul, Turkey; 2 Department of Medical Pharmacology, School of Medicine, Marmara University, Istanbul, Turkey; 3 Department of Pulmonary Medicine, Haydarpasa Numune Training and Research Hospital, Istanbul, Turkey; 4 Department of Basic Sciences / Pharmacology, School of Dentistry, Marmara University, Istanbul, Turkey

**Keywords:** COVID-19, Obstructive airway disease, Drug utilization, Anticholinergics, Glucocorticoids, Utilization pattern

## Abstract

As COVID-19 primarily affects the respiratory system, it may have impacted utilization patterns of drugs used in obstructive airway diseases (DOADs). We examined nationwide DOAD utilization trends before, during, and after pandemic measures. We collected data on DOADs (ATC-Code: R03) between 01.01.2017–28.02.2023 from IQVIA-Turkey. National outpatient sales and prescription projections were converted into consumption data, expressed as defined daily dose per 1,000 inhabitants (DID). We compared mean monthly consumption, costs, and quarterly DOADs use across "before restrictions" (BfR), "during restrictions" (DuR), and "after restrictions" (AfR) periods. We identified 433.5 million DOAD units consumed, costing €3.3 billion; inhaled-DOADs accounted for 73.1%. Mean monthly DOAD consumption remained stable (BfR: 67.8 ± 3.1 DID; DuR: 74.2 ± 12.5 DID; AfR: 74.2 ± 14.6 DID; *p* > 0.05). Inhaled-DOADs exhibited a similar pattern, except the anticholinergics with a significant increase in the DuR (19.4 ± 3.3 DID) compared to the BfR (16.1 ± 2.3 DID, *p* < 0.001). Also inhaled-corticosteroid monotherapy rose significantly between BfR (4.1 ± 0.9 DID) and AfR (5.3 ± 1.3 DID, *p* < 0.05). Montelukast, constituting 76.6% of systemic-DOADs, had higher consumption in AfR (15.0 ± 2.8 DID) than in BfR (11.7 ± 2.2 DID, *p* < 0.001) and DuR (12.9 ± 2.4 DID, *p* < 0.05). Overall DOAD prescriptions declined in DuR and trended upward in AfR but didn’t reach pre-pandemic levels, except for new users of montelukast and long-acting beta agonists. Our study showed increased use of inhaled anticholinergics, glucocorticoid monotherapy, and montelukast within a generally stable overall DOAD utilization. This may reflect varied responses to bronchodilation and anti-inflammatory treatment needs during the pandemic and beyond.

**Supplementary Information:**

The online version contains supplementary material available at 10.1007/s00210-025-04099-7.

## Introduction

The COVID-19 pandemic has prompted substantial shifts in global health paradigms. Key topics of universal discussion during crises like pandemics include access to healthcare and medicines for vulnerable populations, rational disease management, drug waste, and pharmacoeconomic processes (Whaley et al. 2020; Imlach et al. 2021; Suda et al. 2022). Studies on drug use patterns during the pandemic are crucial as they provide data that can be applied globally, reflecting common issues across different regions (Frazer and Frazer 2021; Silva et al. 2023; Vizdiklar et al. 2024).

Obstructive airway diseases (OAD) like asthma and chronic obstructive pulmonary disease (COPD) cause significant morbidity, mortality and health expenditure burden worldwide (Mauer and Taliercio 2020; Reddel et al. 2022; Levy et al. 2023). While respiratory diseases accounted for 9% (COPD 3%) of the reasons for death across all OECD region, Turkey 2021 health statistics data showed surpassing rates with 14% (4% for COPD) (The Ministry of Health of Türkiye 2022). COVID-19, with its severe respiratory symptoms and subsequent course may lead to notable changes in patient attitude and behaviors, especially among those with preexisting respiratory comorbidities (Amirav and Newhouse 2020; Bloom et al. 2021; Dhruve et al. 2022; Silva et al. 2023). These may refer to worsening of symptoms, course or nature of exacerbations, disruptions in healthcare access during lockdowns, and patterns of use of non-routine medications for these conditions (Choi et al. 2021; Bloom et al. 2021; Toennesen et al. 2022). For example, a study in Scotland reported that prescriptions for inhaled corticosteroids surged by 121% from the pandemic’s onset to the start of lockdowns (Davies et al. 2021). Furthermore, as COVID-19 itself is mainly a respiratory disease, pandemic threat may have also impacted utilization patterns of drugs used in obstructive airway diseases (DOADs) (Landete et al. 2022). A comparative exploratory analysis on utilization trend of such drugs could provide valuable insights into the course of disease, its burden on society, healthcare access and pharmacoeconomics during crisis settings. In this study, we aimed to examine the comparative nationwide utilization trend of DOADs before, during and after the COVID-19 pandemic measures.

## Methods

### Study design

In this study, we retrospectively analyzed the changes in the consumption, prescribing and cost of DOADs in Turkey, a country with over 80 million inhabitants (Turkish Statistical Institute 2024), during the periods of changes in restriction practices related to the COVID-19 pandemic. The data used in the study was obtained from IQVIA Turkey. IQVIA is an organization that provides quantitative data on the pharmaceutical market on an international scale (Kirmizi et al. 2022, IQVIA Turkey 2021). For the analyses on drug consumption and cost, unit-based drug outflow data from pharmaceutical warehouses to retail pharmacies across Turkey were used. Accordingly, we collected data from IQVIA Turkey on the number of monthly units sold and the cost of DOADs during the study period which ran from January 1, 2017 to February 28, 2023.

### Data collection

**Fig. 1:**
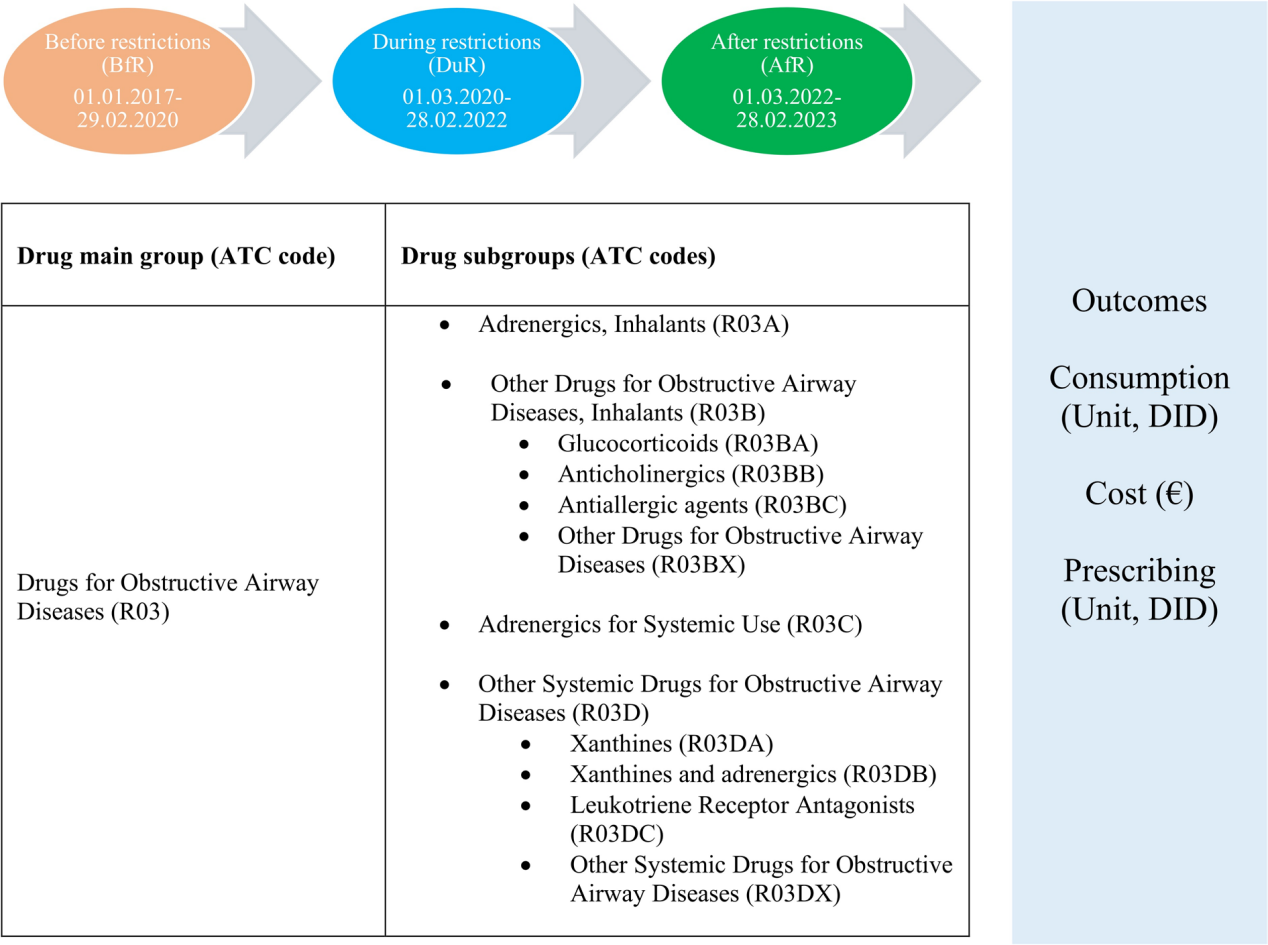
The groups of drugs for obstructive airway diseases evaluated along with the restriction-associated time periods and the outcomes determined in the study. *ATC, Anatomical Therapeutic Chemical*

We selected DOADs as the drugs with ATC-2 code R03 (drugs used in obstructive airway diseases) in the Anatomical Therapeutic Chemical (ATC) classification system established by the World Health Organization (WHO) for the study (Fig. 1). The mean monthly consumption levels of these medicines before restrictions (BfR), during restrictions (DuR) and after restrictions (AfR) were calculated and compared with each other. We determined these periods according to the dates when COVID-19 pandemic-related restrictions were implemented and abolished in Turkey: COVID-19 pandemic-related restriction measures were first applied on 12.03.2020 and these measures were significantly relaxed across the country on 03.03.2022 (Turkish Ministry of Interior 2022). Accordingly, the first 38 months (01.01.2017–29.02.2020) of the study period covering a total of 74 months were designated as BfR, followed by 24 months (01.03.2020–28.02.2022) as DuR and the last 12 months (01.03.2022–28.02.2023) as AfR. Drug consumption expressed in units and DID (defined daily dose per 1000 inhabitants) (WHO Collaborating Centre for Drug Statistics Methodology 2021). In analyses where drug use levels were measured by DID, we did not include the active substances/medicinal products for which DDD values were not defined. Each active substance contained in combined preparations was considered as independent single agents in consumption level analyses and the DDD values determined for their individual preparations were used in the calculations.

In order to analyze the trends in the prescribing of drugs for OADs by physicians, we used the "prescription nationwide projection data" registered in IQVIA. This prescription dataset is created by IQVIA Turkey office collecting prescriptions from 1000 physicians across the country, including the diagnosis and treatment of outpatients for 7 days, and IQVIA Global Statistics Office projecting the diagnosis and treatment information in these prescriptions across Turkey for the quarters of each year. Indication for OAD (International Classification of Diseases-10 (ICD-10) code: J44 (chronic obstructive pulmonary disease [COPD]), J45 (asthma)), the average number of drug units prescribed and the DID values of the DOAD in quartiles corresponding to BfR, DuR and AfR were determined and compared. We analyzed medicines in prescriptions individually, depending on whether they were prescribed for the first time to a patient or re-prescribed to a patient already diagnosed. We further stratified DOADs by their route of delivery, systemic or inhaled, and compared consumption, prescribing (new/repeat) and cost across the BfR, DuR and AfR periods.

Costs related to drug consumption were calculated separately for single active ingredient and combined active ingredient preparations based on average monthly costs in euros. The average monthly costs of each relevant drug in BfR, DuR and AfR were determined and compared using the current euro exchange rates used by the Turkish Ministry of Health for drug pricing.

The study was initiated following the approval of the Istanbul Medipol University Non-Interventional Clinical Research Ethics Committee (13.04.23/342).

### Statistical analysis

Statistical analyses were performed using IBM SPSS 29.0 and GraphPad Prism 10.1 programs. The analyzed data were expressed as number and percentage for categorical variables and mean ± standard deviation for continuous variables. The conformity of continuous variables to normal distribution was evaluated by Shapiro–Wilk normality test. Normally distributed variables were compared by one-way analysis of variance and Tukey's post-hoc test, and non-normally distributed variables were compared by Kruskal–Wallis test and Dunn's post-hoc test. A type 1 error value of less than 5% was accepted as statistical significance.

## Results

The total number and cost of DOADs consumed nationwide over the 74-month study period were 433.5 million units and €3.3 billion, respectively. Inhaled-DOADs accounted for 73.1% of DOADs consumed. Projection data for prescriptions for OADs showed inhaled-DOADs to constitute 77.5% of all DOADs. Consumption and prescribing data for individual DOAD groups is shown at Table 1, with accompanying heatmaps for selected DOADs.

**Table 1:**
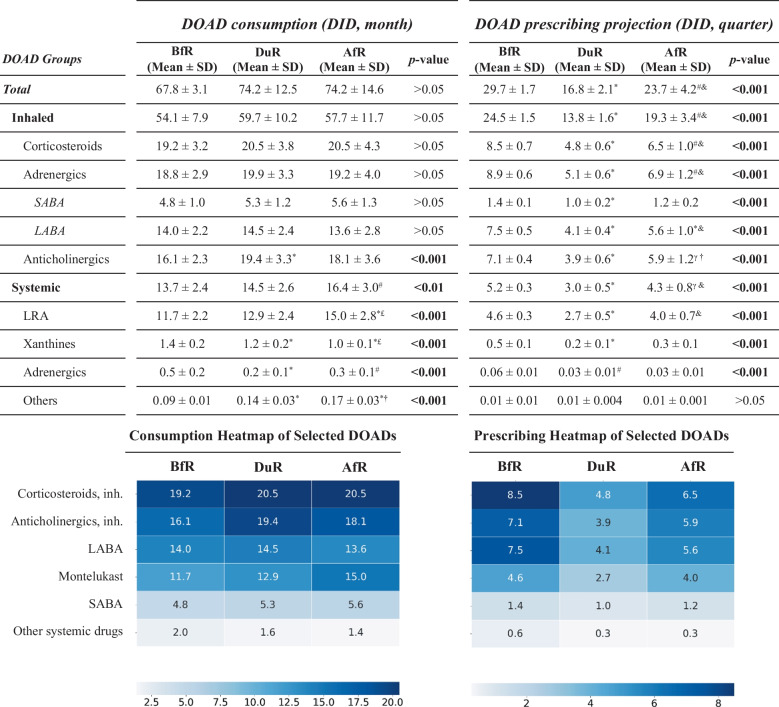
Distribution and comparison of mean values of monthly consumption and quarterly prescribing of DOADs across COVID-19 restriction-associated periods

*DOAD* drugs for obstructive airway diseases; *SABA* Short-Acting Beta Agonist; *LABA* Long-Acting Beta Agonist; *LRA* leukotriene receptor antagonists; *BfR* before restrictions; *DuR* during restrictions; *AfR* after restrictions; *inh.* inhaled. *, *p* < 0.001 vs. BfR; #, *p* < 0.01 vs. BfR; γ, *p* < 0.05 vs. BfR; †, *p* < 0.001 vs. DuR; &, *p* < 0.01 vs. DuR; £, *p* < 0.05 vs. DuR

**Fig. 2:**
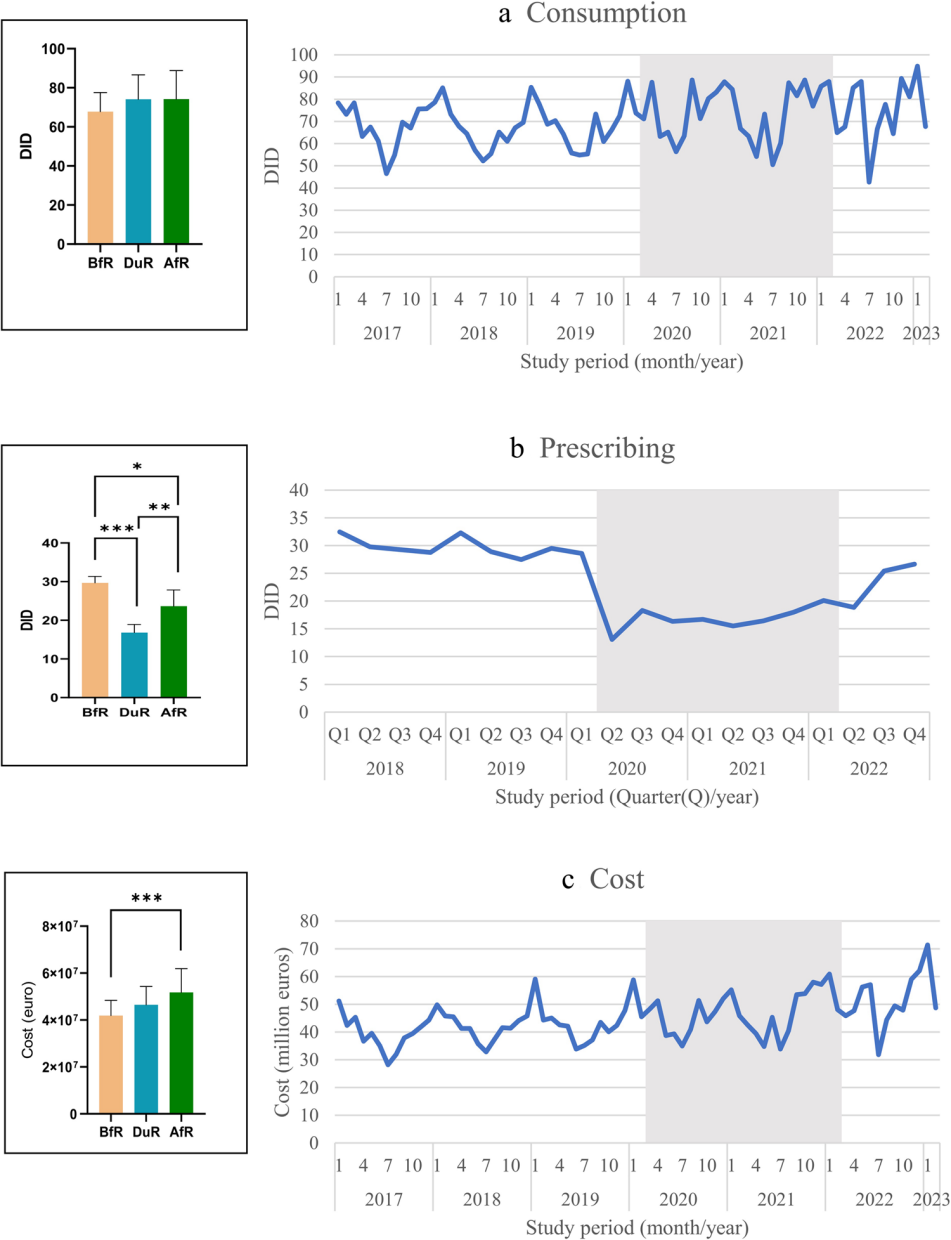
Trends and comparisons of average monthly (**a**) consumption, (**b**) quarterly prescribing and (**c**) average monthly cost of drugs for obstructive airway diseases across COVID-19 restriction-associated periods. *, *p* < 0.05, **, *p* < 0.01, ***, *p* < 0.001

We detected that the mean monthly consumption of DOADs, which was 67.8 ± 3.1 DID in the BfR, reached 74.2 ± 12.5 DID in the DuR and 74.2 ± 14.6 DID in the AfR (*p* > 0.05), (Fig. 2a). The prescribing of these drugs was 29.7 ± 1.7 DID per quarter in the BfR, dropping sharply to 16.8 ± 2.1 DID in the DuR (*p* < 0.001 vs. BfR) and then rebounded in the AfR reaching 23.7 ± 4.2 DID (*p* < 0.01 vs. DuR), significantly below the baseline (*p* < 0.05 vs. BfR), (Fig. 2b). The mean monthly cost of DOADs was €41.9 ± 6.4 million in the BfR and €46.5 ± 7.8 million in the DuR. This cost increased significantly to €51.8 ± 10.1 million in the AfR compared to that in the baseline level (*p* < 0.001), (Fig. 2c, Supplementary Table 1).

The consumption of inhaled-DOADs exhibited a stable pattern across the study periods (54.1 ± 7.9 DID, 59.7 ± 10.2 DID and 57.7 ± 11.7 DID, respectively) (*p* > 0.05). Inhaled anticholinergics constituted 31.0% of inhaled-DOADs, and their consumption showed a significant increase in the DuR (19.4 ± 3.3 DID) compared to that in the BfR (16.1 ± 2.3 DID, *p* < 0.001), and then slightly decreased to 18.1 ± 3.6 DID in the AfR. The sub-analysis of the inhaled-DOAD preparations containing single active substance showed that anticholinergic consumption decreased from 8.2 ± 1.6 DID in BfR to 8.0 ± 1.3 DID in DuR and further to 6.8 ± 1.4 DID in AfR (*p* < 0.05 compared to BfR). In this sub-analysis, the only significant upward trend was observed for inhaled corticosteroids (4.1 ± 0.9 DID, 4.4 ± 1.4 DID and 5.3 ± 1.3 DID, respectively (*p* < 0.05 compared to BfR)).

The prescribing of inhaled-DOADs was similar to the overall DOAD prescribing trend, declining from 24.5 ± 1.5 DID to 13.8 ± 1.6 DID in the DuR (*p* < 0.001 vs. BfR) and then significantly climbing to 19.3 ± 3.4 DID in the AfR (*p* < 0.01 vs. DuR) but below the BfR (*p* < 0.01). When stratified by ongoing vs. new users of inhaled-DOADs, such trend was not observed in the latter, showing rather a comparably stable course of utilization in the BfR (4.8 ± 0.6 DID), DuR (4.1 ± 1.1 DID) and AfR (5.5 ± 0.6 DID) (*p* > 0.05 for pairwise comparisons). In particular, the use of newly prescribed long-acting beta agonist for asthma and inhaled anticholinergic for COPD was significantly increased in the AfR compared to that in the DuR (Table 2). The cost of inhaled-DOADs showed a significantly increasing trend from BfR to the AfR (Supplementary Table 1).

**Table 2:** Distribution and comparison of quarterly prescribing of DOADs across COVID-19 restriction-associated periods, including analysis by the therapeutic indication and new vs. ongoing prescribing

Drug Groups	Indication	New Prescriptions				Ongoing Prescriptions			
		BfR (Mean ± SD)	DuR (Mean ± SD)	AfR (Mean ± SD)	*P*-value	BfR (Mean ± SD)	DuR (Mean ± SD)	AfR (Mean ± SD)	*P*-value
Inhaled adrenergic drugs	Asthma	1.4 ± 1.2	1.2 ± 0.3	1.6 ± 0.2^£^	< 0.05	4.0 ± 0.4	2.3 ± 0.3*	3.0 ± 0.7^#^	** < 0.001**
	COPD	0.5 ± 0.1	0.4 ± 0.1^γ^	0.4 ± 0.1	< 0.05	3.0 ± 0.2	1.2 ± 0.2*	1.7 ± 0.4*^&^	** < 0.001**
	All	1.8 ± 0.2	1.6 ± 0.4	2.1 ± 0.2	> 0.05	7.0 ± 0.5	3.5 ± 0.5*	4.8 ± 1.1	** < 0.001**
SABA	Asthma	0.2 ± 0.05	0.2 ± 0.1	0.2 ± 0.1	> 0.05	0.5 ± 0.1	0.4 ± 0.1^γ^	0.4 ± 0.1	** < 0.05**
	COPD	0.1 ± 0.02	0.1 ± 0.02	0.1 ± 0.02	> 0.05	0.6 ± 0.05	0.3 ± 0.1*	0.4 ± 0.1^£^	** < 0.001**
	All	0.3 ± 0.1	0.3 ± 0.1	0.3 ± 0.1	> 0.05	1.1 ± 0.1	0.7 ± 0.1*	0.9 ± 0.2	** < 0.001**
LABA	Asthma	1.2 ± 0.1	1.0 ± 0.3	1.4 ± 0.1^£^	**< 0.05**	3.5 ± 0.3	2.0 ± 0.3*	2.6 ± 0.6^#^	** < 0.001**
	COPD	0.3 ± 0.1	0.3 ± 0.1	0.3 ± 0.04	> 0.05	2.4 ± 0.2	0.9 ± 0.1*	1.3 ± 0.3*^£^	** < 0.001**
	All	1.5 ± 0.2	1.3 ± 0.3	1.7 ± 0.2^£^	** < 0.05**	6.0 ± 0.5	2.8 ± 0.4*	3.9 ± 0.9	** < 0.001**
Inhaled corticosteroids	Asthma	1.4 ± 0.2	1.2 ± 0.4	1.7 ± 0.2	> 0.05	4.1 ± 0.5	2.3 ± 0.3*	3.1 ± 0.8^#^	** < 0.001**
	COPD	0.3 ± 0.1	0.2 ± 0.1	0.3 ± 0.03	> 0.05	2.6 ± 0.2	1.0 ± 0.1*	1.5 ± 0.3*^£^	** < 0.001**
	All	1.7 ± 0.2	1.5 ± 0.4	2.0 ± 0.2	> 0.05	6.7 ± 0.6	3.3 ± 0.4*	4.6 ± 1.0*^&^	** < 0.001**
Inhaled anticholinergic drugs	Asthma	0.2 ± 0.05	0.2 ± 0.1	0.3 ± 0.1	> 0.05	1.4 ± 0.3	0.8 ± 0.1^#^	1.3 ± 0.4	** < 0.001**
	COPD	1.0 ± 0.2	0.8 ± 0.2	1.2 ± 0.1^£^	** < 0.05**	4.5 ± 0.1	2.1 ± 0.3*	3.2 ± 0.8*^†^	** < 0.001**
	All	1.2 ± 0.2	1.0 ± 0.3	1.5 ± 0.1^£^	** < 0.05**	5.9 ± 0.4	2.9 ± 0.4*	4.5 ± 1.2	** < 0.001**
Systemic LRA	Asthma	1.3 ± 0.1	0.9 ± 0.3^#^	1.4 ± 0.1^£^	** < 0.01**	3.1 ± 0.2	1.8 ± 0.3*	2.5 ± 0.6	** < 0.001**
	COPD	0.03 ± 0.01	0.02 ± 0.01	0.03 ± 0.004	> 0.05	0.2 ± 0.1	0.1 ± 0.1^#^	0.1 ± 0.1	** < 0.01**
	All	1.3 ± 0.1	0.9 ± 0.3^#^	1.4 ± 0.1^£^	** < 0.01**	3.3 ± 0.2	1.8 ± 0.3*	2.6 ± 0.7^γ&^	**< 0.001**
Systemic xanthines	Asthma	0.03 ± 0.01	0.01 ± 0.01	0.02 ± 0.01	> 0.05	0.2 ± 0.03	0.1 ± 0.02*	0.1 ± 0.04^#^	** < 0.001**
	COPD	0.1 ± 0.01	0.04 ± 0.01	0.05 ± 0.004	> 0.05	0.3 ± 0.1	0.1 ± 0.04*	0.2 ± 0.02^£^	** < 0.001**
	All	0.1 ± 0.02	0.1 ± 0.02	0.1 ± 0.01	> 0.05	0.4 ± 0.1	0.2 ± 0.1*	0.3 ± 0.1^#^	** < 0.001**
Systemic adrenergic drugs	Asthma	0.03 ± 0.01	0.01 ± 0.004*	0.01 ± 0.01^#^	** < 0.01**	0.02 ± 0.01	0.01 ± 0.001	< 0.01^γ^	** < 0.05**
	COPD	< 0.01	< 0.01	0.01 ± 0.01	> 0.05	0.01 ± 0.005	0.01 ± 0.01	< 0.01	> 0.05
	All	0.03 ± 0.01	0.01 ± 0.005*	0.02 ± 0.01^£^	** < 0.01**	0.03 ± 0.01	0.01 ± 0.01	0.01 ± 0.005^#^	** < 0.01**
Systemic others	Asthma	0.01 ± 0.01	0.01 ± 0.001	< 0.01	> 0.05	0.01 ± 0.004	0.01 ± 0.004	0.01 ± 0.001	> 0.05
	COPD	< 0.01	0	0	> 0.05	< 0.01	0^γ^	0	** < 0.01**
	All	0.01 ± 0.01	0.01 ± 0.004	0.01 ± 0.001	> 0.05	0.01 ± 0.01	0.01 ± 0.004	0.01 ± 0.001	> 0.05

*COPD* chronic obstructive pulmonary disease; *DOAD* drugs for obstructive airway diseases; *SABA* Short-Acting Beta Agonist; *LABA* Long-Acting Beta Agonist; *LRA* leukotriene receptor antagonists. *BfR* before restrictions, *DuR* during restrictions, *AfR* after restrictions. *, *p* < 0.001 vs. BfR; #, *p* < 0.01 vs. BfR; γ, *p* < 0.05 vs. BfR; †, *p* < 0.001 vs. DuR; &, *p* < 0.01 vs. DuR; £, *p* < 0.05 vs. DuR

Systemic-DOAD consumption increased significantly from 13.7 ± 2.4 DID in BfR to 16.4 ± 3.0 DID in AfR (*p* < 0.01), with 14.5 ± 2.6 DID in DuR (*p* > 0.05 for pairwise comparisons). Montelukast constituted 76.6% of the systemic-DOADs. Montelukast consumption was significantly higher in the AfR (15.0 ± 2.8 DID) than that in the BfR (11.7 ± 2.2 DID, *p* < 0.001) and DuR (12.9 ± 2.4 DID, *p* < 0.05). The other drug groups in the systemic-DOAD exhibited a significant decline overall (*p* < 0.001). The prescribing of systemic-DOADs significantly fell from 5.2 ± 0.3 DID in the BfR to 3.0 ± 0.5 DID in the DuR (*p* < 0.001), before recovering to 4.3 ± 0.8 DID in the AfR (*p* < 0.05 vs. BfR, *p* < 0.01 vs. DuR). The only exception was new users of montelukast, which restored pre-pandemic use (1.3 ± 0.1 DID) in the AfR (1.4 ± 0.1 DID), significantly higher than that in the DuR (0.9 ± 0.3 DID, *p* < 0.05).

## Discussion

Our study provides a detailed overview of trends in the use of medicines for treating OADs across a large national population during the COVID-19 pandemic, focusing on how these trends were influenced by different periods of restriction. While the overall consumption of all drug groups exhibited relatively stable pattern, we observed notable changes during pandemic for specific subgroups, including those containing inhaled anticholinergics and inhaled glucocorticoid monotherapy. Our findings, together with the post-pandemic increase in systemic drugs, driven by montelukast, highlight the diverse responses in DOAD use during and after the pandemic.

During the COVID-19 pandemic, changes in treatment patterns for obstructive diseases, which primarily affect the respiratory system, were inevitable. Literature suggests that conditions like asthma attacks, which dictate the use of medications for obstructive diseases, have deviated from their real-world course due to measures like isolation and mask-wearing (Amirav and Newhouse 2020; Choi et al. 2021; Bloom et al. 2021; Suda et al. 2022; Toennesen et al. 2022). For example, an international study using IQVIA MIDAS data found a nearly 30% surge in sales of respiratory system medicines following the pandemic announcement, followed by a 13.6% drop between March and August 2020 (Suda et al. 2022). This surge is attributed to drug stockpiling, a phenomenon noted in various studies on respiratory drugs worldwide (Frazer and Frazer 2021; Silva et al. 2023). It is also noteworthy that similar patterns are also observed in non-respiratory medications (Suda et al. 2022; Selke Krulichová et al. 2022; Vizdiklar et al. 2024). In our study, the overall consumption of DOADs and inhaled-DOADs did not significantly fluctuate across different periods. This stability suggests that, despite initial stockpiling driven by panic, the reduced need for medication due to fewer OAD attacks during restrictions may have balanced out consumption (Shah et al. 2021). Supporting this, our prescription data showed a sharp decline between the BfR and DuR periods, followed by a significant increase from DuR to AfR. Notably, the utilization in the AfR (23.7 DID) remained below the BfR (29.7 DID). This may be related to the stockpiling behavior at the beginning of the pandemic, which resulted in patients consuming excess medicines, as well as the benefit of sterile conditions during the lockdown period and the availability of reported medicines without a prescription in Turkey. Indeed, the significant decrease in repeat prescriptions from BfR to DuR suggests that patients with OAD needed less medication or obtained it without visiting the physician, consistent with other studies (Shah et al. 2021; Toennesen et al. 2022; Silva et al. 2023). The rise in prescriptions of new cases between DuR and AfR may be explained by incident OAD diagnoses after COVID-19. A meta-analysis of 196 studies reported that 45% of COVID patients had at least one unresolved symptom at 4 months (O’Mahoney et al. 2023). In addition, a nationwide Scottish study showed the absolute prevalence of long-COVID as 10.4%, with near a quarter complaining of breathlessness at 18 months (Hastie et al. 2023). In fact, a recent population-based cohort reported an increasing risk of developing respiratory diseases up to 24-month follow-up (Meng et al. 2024), consistent with the ascending trend in DOAD-containing prescriptions of new cases in our study.

Corticosteroids are frequently used agents during the pandemic period, not only in patients with OADs, as they reduce mortality in hospitalized patients and inhibit the inflammatory response leading to multiple organ failure (Shuto et al. 2020; Li et al. 2021). Studies have also demonstrated that routine use of inhaled corticosteroids in patients with obstructive airways may be protective against pandemic-related problems (Schultze and Douglas 2021; Bafadhel et al. 2022). In addition, asthma patients tend to seek medical care more often with a lower threshold of physicians for intervention (Bloom 2023). Concurrently, adherence to inhaled corticosteroid in asthma patients was reported to improve during the pandemic (Dhruve et al. 2022). This may be due to patients prioritizing preventive measures and early treatment of respiratory symptoms because of lingering concerns about COVID-19. The rising interest in corticosteroid preparations was also visible in our study. It is noteworthy that the use of inhaled-DOADs containing single corticosteroids increased in all periods and the consumption in AfR was significantly higher than in BfR. The discrepancy between consumption and prescription data suggests that inhaled corticosteroids are often prescribed for conditions beyond obstructive respiratory diseases, potentially including pandemic-related issues.

COPD is more common in the geriatric population than asthma, often with other comorbid conditions (Agustí et al. 2023). It is reported in the literature that the incidence of pneumonia and hospitalization of patients with COPD is higher if they have COVID-19 (Graziani et al. 2020). Furthermore, these patients had higher odds of developing long COVID (Erinoso et al. 2024). Notably, our study showed a significant increase in the consumption of inhaled anticholinergics, a key treatment for COPD patients. This spike could be attributed to several factors. Consistent with existing literature (Silva et al. 2023), some COPD patients may have stockpiled these medications out of pandemic-induced panic. Additionally, COVID-19 patients with COPD might have required extra bronchodilation, mirroring the use of corticosteroids for managing COVID-19. In fact, a German cohort of COPD patients reported clinical deterioration despite higher adherence to protective measures (Kahnert et al. 2021). Our findings also showed 50% increased new prescriptions of anticholinergics in COPD management. Furthermore, the limited access to healthcare services during the pandemic might have amplified the need for additional bronchodilation.

In this study, in contrast to the decrease in other systemic DOADs, the increased consumption of montelukast, which constitutes the majority of the systemic-DOAD group, was remarkable, particularly after the pandemic. As confirmed by our prescription data by diagnosis, montelukast is mainly used for asthma owing to its anti-inflammatory properties and potential role in preventing bronchial remodeling (Paggiaro and Bacci 2011). A population-based cohort study reported elevated asthma risk among COVID patients still after 2 years (Meng et al. 2024). This might partly explain the post-pandemic increase in new users of montelukast in our study, which was also the case for new users of long-acting beta agonists. On the other hand, beta agonist and inhaled glucocorticoid prescriptions for pre-existing asthma remained lower in AfR than in BfR. This might be modestly contributed by increased utilization of montelukast, which was shown to diminish beta agonist use (Vaquerizo 2003) and help to spare the use of inhaled corticosteroids (Ye et al. 2015). Another reason accounting for increased consumption of montelukast could be its effectiveness in relieving cough variant asthma (Xu et al. 2023), as a large series showed cough prevalence of 13.1% at 6 months of COVID-19 (Groff et al. 2021). Although montelukast failed to prove efficacy in postinfectious cough in a randomized controlled trial (Wang et al. 2014), weak evidence showed improvement in post-COVID cough duration and severity (Mohamed Hussein et al. 2022). Our findings indicate the need for conducting both qualitative and quantitative studies to investigate the reasons behind and provide rationale for increased montelukast utilization, contrary to the comparably stable course of inhaled-DOAD consumption.

It is known that medicines used in the treatment of obstructive diseases are a significant cost factor for the healthcare system, especially inhaled-DOADs (Tavakoli et al. 2019; Levy et al. 2023). It is also noteworthy that prescription data has not returned to BfR-era levels even in the AfR, while costs have followed an increasing pattern. This may be associated with increasing inflation and population-related market growth, as well as the prescription of these drugs for COVID-19-related reasons other than OAD. A detailed analysis of the rational management of these diseases, not only in pharmacotherapy but also in pharmacoeconomics, will provide important insights for stakeholders.

Our study's findings should be viewed with an understanding of its limitations. The drug consumption data represents sales from pharmaceutical warehouses to community pharmacies, but it assumes that these medications were actually used by patients. Additionally, there is no patient-specific data such as demographics, dosage, and comorbidities. Lack of knowledge and skills in inhaled drug use among patients also affects adherence (Chrystyn et al. 2019), and this aspect couldn't be explored due to restricted access to healthcare during the pandemic, which may have influenced consumption habits. The prescription projection data, produced quarterly by IQVIA Turkey, might miss monthly fluctuations. Furthermore, DOADs can also be used for COVID-19 treatment, but the lack of a database showing COVID-19-related drug use precluded pandemic-related comparisons in this study. Finally, advanced statistical methods such as machine learning-based predictive modeling and feature selection techniques have been increasingly utilized in biomedical data analysis to infer disease occurrence (Banappagoudar et al. 2025; Jena et al. 2025). However, the structure and characteristics of our dataset of drug use did not allow for the application of such methods, though the uniqueness of the indications of the drugs evaluated could give valuable insights about the epidemiology of obstructive airway diseases during study periods, partly addressing such limitation.

## Conclusions

In conclusion, this study provides critical insights into the utilization of DOADs, where the use of inhaled anticholinergics, glucocorticoid monotherapy, and montelukast demonstrated increased consumption over the rest during and/or after the pandemic with large population data. This pattern underscores the varied responses to the need for bronchodilation and anti-inflammatory treatments during critical periods of the pandemic and beyond. The overall stable course of consumption despite reduced prescription levels during the pandemic might be attributable to expedited medication access and various patient behaviors during health crises. The findings suggest a complex interplay between disease management, prescription behaviors, and external factors such as healthcare access and public health measures. However, the stability in overall DOAD use despite increases in specific drug categories calls for further investigation into patient behaviors, stockpiling tendencies, and possible shifts in disease prevalence or diagnosis rates due to the pandemic. Our findings can inform the management of chronic diseases and contribute to national and international evaluations in future public health planning.

## Supplementary Information

Below is the link to the electronic supplementary material.

Supplementary file1 (DOCX 17 KB)

## Data Availability

All source data for this work (or generated in this study) are available upon reasonable request.
