# Supplementary material for: Outpatient utilization trend of bronchodilator and anti-inflammatory agents in the pandemic and beyond
Source: Naunyn Schmiedebergs Arch Pharmacol. 2025 Apr 8;398(9):12647–56. doi: 10.1007/s00210-025-04099-7 (PMC12449374; doi:10.1007/s00210-025-04099-7)
Supplement: Supplementary file 1 — Supplementary file1 (DOCX 17 KB) [file 210_2025_4099_MOESM1_ESM.docx]

**Supplementary Table 1.** Distribution and comparison of the average values (euros) of monthly consumption of drugs for obstructive airway diseases across COVID-19 restriction-associated periods.

| **Preparations** | **Drug groups** | **BfR**  **(Mean ± SD)** | **DuR**  **(Mean ± SD)** | **AfR**  **(Mean ± SD)** | **P-value** |
| --- | --- | --- | --- | --- | --- |
| **Combined preparations** | Adrenergics with corticosteroids | 12.4 ± 2.0 | 13.9 ± 2.3^γ^ | 13.9 ± 2.7 | <0.05 |
|  | Adrenergics with anticholinergics with or without corticosteroids | 3.6 ± 1.1 | 5.1 ± 0.9^*^ | 5.5 ± 1.4^*^ | <0.001 |
|  | Montelukast combinations | 3.5 ± 0.6 | 3.8 ± 0.7 | 5.3 ± 1.2^*†^ | <0.001 |
|  | **Total** | 19.5 ± 2.9 | 22.8 ± 3.6^#^ | 24.7 ± 5.1^*^ | <0.001 |
| **Single active ingredient preparations** | **Inhaled Drugs** | 14.7 ± 2.7 | 14.4 ± 3.4 | 16.4 ± 4.0 | >0.05 |
|  | Glucocorticoids | 6.4 ± 1.3 | 6.8 ± 2.0 | 8.0 ± 2.3^γ^ | <0.05 |
|  | Adrenergics | 3.7 ± 1.0 | 3.1 ± 1.2^γ^ | 4.4 ± 1.3^&^ | <0.01 |
|  | Anticholinergics | 4.6 ± 0.8 | 4.5 ± 0.7 | 4.0 ± 0.9 | >0.05 |
|  | **Systemic Drugs** | 7.7 ± 1.3 | 9.2 ± 1.5^*^ | 10.8 ± 1.8^*£^ | <0.001 |
|  | Leukotriene receptor antagonists | 2.7 ± 1.3 | 2.4 ± 1.5 | 2.4 ± 1.8 | >0.05 |
|  | Xanthines | 0.4 ± 0.7 | 0.4 ± 0.5^#^ | 0.4 ± 0.6^*^ | <0.001 |
|  | Adrenergics | 0.4 ± 0.1 | 0.2 ± 0.1^*^ | 0.3 ± 0.1 | <0.001 |
|  | Others | 4.2 ± 0.2 | 6.3 ± 1.3^*^ | 7.7 ± 1.4^*†^ | <0.001 |
|  | **Total** | 22.4 ± 3.7 | 23.7 ± 4.6 | 27.1 ± 5.2^#^ | <0.01 |
| **All preparations** | Inhaled drugs | 30.7 ± 4.9 | 33.4 ± 6.0 | 35.7 ± 7.7^γ^ | <0.05 |
|  | Systemic drugs | 11.2 ± 1.8 | 13.1 ± 2.2^#^ | 16.0 ± 2.8^*†^ | <0.001 |
|  | **Total** | 41.9 ± 6.4 | 46.5 ± 7.8 | 51.8 ± 10.1^*^ | <0.001 |

BfR, before restrictions of COVID-19 (01.01.2017-29.02.2020); DuR, during restrictions of COVID-19 (01.03.2020-28.02.2022); AfR, after restrictions of COVID-19 (01.03.2022-28.02.2023). *, p<0.001 vs. BfR; #, p<0.01 vs. BfR; γ, p<0.05 vs. BfR; †, p<0.001 vs. DuR; &, p<0.01 vs. DuR; £, p<0.05 vs. DuR
